# Survival rate in nasopharyngeal carcinoma improved by high caseload volume: a nationwide population-based study in Taiwan

**DOI:** 10.1186/1748-717X-6-92

**Published:** 2011-08-11

**Authors:** Ching-Chih Lee, Tze-Ta Huang, Moon-Sing Lee, Yu-Chieh Su, Pesus Chou, Shih-Hsuan Hsiao, Wen-Yen Chiou, Hon-Yi Lin, Sou-Hsin Chien, Shih-Kai Hung

**Affiliations:** 1 Department of Otolaryngology, Buddhist Dalin Tzu Chi General Hospital, Chiayi, Taiwan; 2 Department of Oral and Maxillofacial Surgery, Buddhist Dalin Tzu Chi General Hospital, Chiayi, Taiwan; 3 Department of Radiation Oncology, Buddhist Dalin Tzu Chi General Hospital, Chiayi, Taiwan; 4 Department of Hematology Oncology, Buddhist Dalin Tzu Chi General Hospital, Chiayi, Taiwan; 5 Division of Plastic Surgery, Department of Surgery, Buddhist Dalin Tzu Chi General Hospital, Chiayi, Taiwan; 6 School of Medicine, Tzu Chi University, Hualien, Taiwan; 7 Community Medicine Research Center and Institute of Public Health, National Yang-Ming University, Taipei, Taiwan

## Abstract

**Background:**

Positive correlation between caseload and outcome has previously been validated for several procedures and cancer treatments. However, there is no information linking caseload and outcome of nasopharyngeal carcinoma (NPC) treatment. We used nationwide population-based data to examine the association between physician case volume and survival rates of patients with NPC.

**Methods:**

Between 1998 and 2000, a total of 1225 patients were identified from the Taiwan National Health Insurance Research Database. Survival analysis, the Cox proportional hazards model, and propensity score were used to assess the relationship between 10-year survival rates and physician caseloads.

**Results:**

As the caseload of individual physicians increased, unadjusted 10-year survival rates increased (*p *< 0.001). Using a Cox proportional hazard model, patients with NPC treated by high-volume physicians (caseload ≥ 35) had better survival rates (*p *= 0.001) after adjusting for comorbidities, hospital, and treatment modality. When analyzed by propensity score, the adjusted 10-year survival rate differed significantly between patients treated by high-volume physicians and patients treated by low/medium-volume physicians (75% *vs*. 61%; *p *< 0.001).

**Conclusions:**

Our data confirm a positive volume-outcome relationship for NPC. After adjusting for differences in the case mix, our analysis found treatment of NPC by high-volume physicians improved 10-year survival rate.

## Introduction

The fact that increased caseload is associated with better patient outcomes has been noted for three decades in many areas of health care, including acute myocardial infarction, many types of high-risk surgeries, and cancer treatment [1,2]. The "practice makes perfect" hypothesis may be valid for certain procedures such as open-heart and vascular surgery and "selective referral" may in part account for this phenomenon [3,4]. However, such a positive volume-outcome relationship is not well validated for other procedures. Only a few studies have examined the effect of physician caseload on treatment outcome for head and neck cancers [5,6].

Taiwan has a high incidence of nasopharyngeal carcinoma (NPC): the annual incidence rate is 6.17 per 100,000 as compared with < 1 per 100,000 in Western countries [7]. Radiotherapy or concurrent chemoradiotherapy (CCRT) is the principal treatment because NPC is anatomically inaccessible and highly sensitive to radiotherapy and chemotherapy [8].

Previous volume-outcome studies have shown improved treatment outcome in breast cancer, oral cancer, esophageal cancer, radical prostatectomy, and nephrectomy [5,9-11]. However, there is scant information on the volume-outcome relationship for NPC. The purpose of this study was to examine the relationship between physician caseload and survival rate in NPC using population-based data.

In most previous studies on the association between caseload and outcome, a Cox proportional hazards model or logistic regression was routinely used, raising the possibility that selection bias might still exist. Therefore, we evaluated the association between physician caseload and survival rate using population-based data, Cox regression analysis, and propensity score to minimize the effect of selection bias.

## Patients and methods

The database contained a registry of contracted medical facilities, a registry of board-certified physicians, and monthly claims summary for all inpatient claims. Because these were de-identified secondary data, this study was exempt from full review by the internal review board.

### Patients and study design

We used data for the years 1998 to 2008 from the National Health Insurance (NHI) Research Database, which contains data on all covered medical benefit claims for over 23 million people in Taiwan (approximately 97 percent of the island's population).

All patients with NPC (International Classification of Disease, Ninth Revision, Clinical Modification codes 147.0-147.9) who received curative treatment by radiotherapy or chemoradiotherapy between the years 1998 and 2000 were included. Patients with unclear treatment modality and incomplete physician data or treated by physicians with a very small caseload (less than 4 cases within 3 years) were excluded. Finally, 1225 patients treated by 98 radiation oncologist during this period were included.

Physicians were further sorted by their total patient volume using the unique physician identifiers in this database and by their caseload of NPC patients. The volume category cutoff points (high, medium, and low) were determined by sorting the 1225 patients into 3 groups of approximately equal size (4-16 cases [low], 17-34 cases [medium], and ≧35 cases [high]) as previously described [5,12,13].

These NPC patients were then linked to the death data extracted from the records covering the years 1998 to 2008.

#### Measurements

The key dependent variable of interest was the 10-year survival rate. The key independent variables were the NPC caseloads (low, medium, or high). Other physician characteristics included age (≦40, 41-50, ≧51 years) and gender. Patient characteristics included age, gender, geographic location, treatment modality, severity of disease, and enrollee category (EC). The disease severity in each patient was assessed using the modified Charlson Comorbidity Index score, which has been widely used in recent years for risk adjustment in administrative claims data sets [14].

This study used EC as a proxy measure of socioeconomic status, which is an important prognostic factor for cancer patients [15,16]. Patients with NPC were classified into 4 subgroups: EC 1 (civil servants, full-time or regular paid personnel with a government affiliation), EC 2 (employees of privately owned institutions), EC 3 (self-employed individuals, other employees, and members of farmers' or fishermen's associations), and EC 4 (veterans, low-income families, and substitute service draftees) [17].

The hospitals were categorized by ownership (public, not-for-profit or for-profit), geographic location (Northern, Central, Southern, and Eastern Taiwan), and hospital type (medical center, regional hospital, and district hospital).

#### Statistical analysis

The SAS statistical package (version 9.2; SAS Institute, Inc., Cary, N.C.) and SPSS (version 15, SPSS Inc., Chicago, IL, USA) were used for data analysis. A two-sided value of *p *< 0.05 was used to determine statistical significance.

The cumulative 10-year survival rates and the survival curves of each group were compared by the log-rank test. Survival was measured from the time of NPC diagnosis to the time of death. Cox proportional regression model and survival analysis with propensity score stratification were used to compare outcomes between different caseload size groups.

##### (1) Cox proportional hazards model

The Cox proportional regression model was used to evaluate the effect of caseload on survival rate after adjusting for hospital type, surgeon characteristics, and patient demographics.

##### (2) Propensity score

Propensity analysis was used to reduce the effect of selection bias on our hypothesis as described by Rosenbaum and Rubin [18-20]. Propensity score stratification replaces the many confounding factors that may be present in an observational study with a variable of these factors. To calculate the propensity score, patient characteristics in this study were entered into a logistic regression model predicting selection for high-volume surgeons. These characteristics included year in which the patient was diagnosed, age, gender, Charlson Comorbidity Index score, geographic area of residence, enrollee category, and treatment modality. The study population was then divided into five discrete strata on the basis of propensity score. The effect of caseload assignment on 10-year survival rate was analyzed within each quintile. The Mantel-Haenszel odds ratio was calculated in addition to the Cochran-Mantel-Haenszel χ^2 ^statistic.

## Results

A total of 423 patients (35%) died out of 1225 patients who underwent curative treatment between 1998 and 2000. A total of 98 radiation oncologists were included. The characteristics of the physicians and patients are summarized in Tables 1 and 2. The majority of the patients were male (72%). Patients in the high-volume physician group were more likely to undergo radiotherapy, reside in Northern Taiwan, have lower comorbidity score, and better enrollee category than their counterparts in other groups. There were 74 radiation oncologists (76%) in the low-volume group, 17 physicians (17%) in the medium-volume group, and 7 (7%) physicians in the high-volume group. The mean age of all physicians was 40 ± 12 years. There was no significant difference in age between these three caseload groups (*p *= 0.507).

**Table 1:** Patient Characteristics in Different Caseload Groups (*n *= 1225)

	NPC caseload group			
		
Variable	Low (4-16) (*n *= 424)	Medium (17-34) (*n *= 394)	High (35-152) (*n *= 407)	*p *
Age				0.037
35-44 years	136(32)	90(23)	103(25)	
45-54 years	118(28)	143(36)	145(36)	
55-64 years	93(22)	100(25)	99(24)	
65-74 years	59(14)	51(13)	48(12)	
≧ 75 years	18(4)	10(3)	12(3)	
Gender				0.389
Male	316(75)	285(72)	286(70)	
Female	108(25)	109(28)	121(30)	
Charlson Comorbidity Index score				< 0.001
< 4	216(51)	229(58)	274(67)	
≧4	208(49)	165(42)	133(33)	
Treatment modality				< 0.001
Radiotherapy	278(66)	271(69)	322(79)	
Chemoradiotherapy	146(34)	123(31)	85(21)	
Geographic location				< 0.001
North	266(63)	240(61)	317(78)	
Central	93(22)	61(15)	43(11)	
Southern and Eastern	65(15)	93(24)	47(11)	
Enrollee category				0.008
EC 1-2	168(40)	133(34)	183(45)	
EC 3	181(43)	172(44)	164(40)	
EC 4	75(18)	89(23)	60(15)	

Values are given as number (percentage).

**Table 2:** Physician Characteristics (*n *= 98)

	Physician caseload group			
		
Variable	Low (4-16)	Medium (17-34)	High (35-152)	*p*
Total no. physicians	74	17	7	
Age(year)				0.507
Mean ± SD	39 ± 13	39 ± 11	45 ± 13	
Gender				0.832
Male	65(88)	14(82)	6(86)	
Female	9(12)	3(18)	1(14)	
Caseload				< 0.001
Mean ± SD	6 ± 5	24 ± 6	62 ± 45	

Values are given as number (percentage).

Abbreviations: SD = standard deviation.

### Analysis using a Cox proportional hazards model

**Figure 1:**
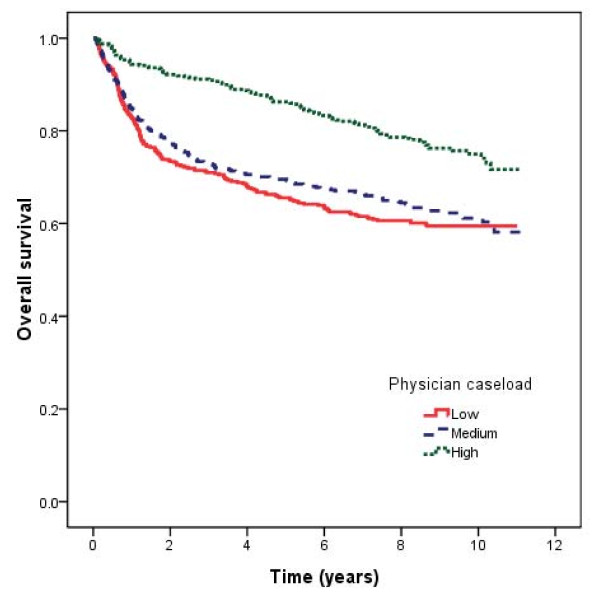
**Nasopharyngeal carcinoma survival rates by physician caseload**.

The 10-year survival rate, by physician caseload group, is shown in Figure 1. The 10-year survival rates were 75%, 61%, and 60% for low-, medium-, and high-volume surgeons, respectively (*p *< 0.001). Table 3 shows the adjusted hazard ratios calculated using the Cox proportional hazards regression model after adjusting for patient comorbidities, hospital type, and treatment modality. The positive association between survival and physician caseload remained statistically significant in multivariate analysis. Patients treated by high-volume physicians had better survival rates (hazard ratio [HR] = 0.6; 95% confidence interval [CI], 0.45-0.78; *p *< 0.001) after adjust other factors.

**Table 3:** Nasopharyngeal Carcinoma Survival Rate and Adjusted Hazard Ratios by Physician Caseload Groups and the Characteristics of the Patients and Providers (*n *= 1225)

Variable	Adjusted hazard ratio	95% CI	*p*
Physician characteristics			
Physician volume			
Low (3-17)	1		
Medium (17-53)	0.884	0.70-1.16	0.884
High (54-130)	0.60	0.45-0.78	< 0.001
Physician age			
≦40 years	1		
41-50 years	1.22	0.97-1.52	0.086
≥51 years	0.78	0.59-1.02	0.073
Hospital characteristics			
Hospital ownership			
Public	1		
Non-for-profit	1.11	0.87-1.42	0.414
For-profit	0.94	0.65-1.36	0.746
Hospital level			
Medical center	1		
Regional hospital	0.88	0.68-1.16	0.368
District hospital	1.25	0.77-2.03	0.376
Patient characteristics			
Patient gender			
Female	1		
Male	0.93	0.75-1.15	0.509
Patient age			
35-44 years	1		
45-54 years	1.15	0.89-1.49	0.277
55-64 years	1.10	0.83-1.45	0.507
65-74 years	1.12	0.81-1.56	0.488
≧ 75 years	0.88	0.48-1.51	0.675
Charlson Comorbidity Index score			
< 4	1		
≧4	1.28	1.04-1.56	0.018
Treatment modality			
Radiotherapy	1		
Chemoradiotherapy	1.03	0.82-1.29	0.784
Geographic location			
North	1		
Central	1.18	0.90-1.55	0.242
Southern and Eastern	1.30	1.00-1.70	0.051
Enrollee category			
EC 1-2	1		
EC 3	1.35	0.71-2.55	0.358
EC 4	1.04	0.86-1.26	0.698

95% CI, 95% confidence interval.

### Analysis using propensity scores

Patients were stratified by propensity score and the effect of physician caseload on survival was assessed. The population was stratified into propensity quintiles as previously described. Table 4 shows survival rates for both caseload groups after stratification. The percentage of patients treated by low/medium-volume physicians decreased from the first propensity quintile to the fifth as predicted by the propensity model. In each of the five strata, patients treated by high-volume physicians had a higher 10-year survival rate. The *p *value for the Cochran-Mantel-Haenszel statistic for the difference in survival between patients treated by low/medium- and high-volume physicians, while controlling for propensity score, was < 0.001, with fewer patients dying who were treated by high-volume physicians (adjusted odds ratio = 0.54, 95% CI, 0.41-0.7). The adjusted 10-year survival rates for low/medium- and high-volume physicians were 61% and 75% (*p *< 0.001).

**Table 4:** 10-year survival of NPC patients in different propensity score strata; low/medium-volume *vs. *high-volume physicians^a^

Propensity score stratum	Low/medium-volume physician group			High-volume physician group			*p*
		
	**No**.	% of stratum	Survival rate (%)	**No**.	% of stratum	Survival rate (%)	
1	193	79	56	51	21	75	0.004
2	191	78	59	52	22	74	0.029
3	173	70	57	74	30	75	0.013
4	145	58	64	104	42	76	0.021
5	116	48	69	126	52	76	0.28
Total	818		61	407	33	75	< 0.001

a. Stratum 1 had the strongest propensity for low/medium physicians; stratum 5, for high-volume physicians.

b. Conchran-Mantel-Haenszel statistics; adjusted odds ratio = 0.54, 95% confidence interval = 0.41-0.70.

In summary, NPC patients treated by high-volume physicians had better survival. The robustness of this result was demonstrated by two different multivariate analyses, the Cox proportional regression model and stratification by propensity score.

## Discussion

Using a Cox proportional hazards model and propensity score, the relative benefit of treatment by high-volume physicians over low/medium-volume physicians was evaluated in NPC. After controlling for patient characteristics and other variables in the Cox proportional regression model, the adjusted hazard ratio was 0.6 for high-volume physicians, indicating that patients with NPC treated by high-volume physicians had a lower risk of death and were more likely to live longer. When analyzed by propensity score, the adjusted 10-year survival rate was 75% for patients treated by high-volume physicians and 61% for patients treated by low/medium-volume physicians. Moreover, fewer patients treated by high-volume physicians died. The results of both forms of analyses led to the conclusion that the 10-year survival rates for patients with NPC treated by high-volume physicians were significantly better.

Previous studies have evaluated the benefits of high hospital and physician volume on the outcomes of cancer treatment. In head and neck cancer, Lin et al. reported that physician volume (not hospital volume) was associated with oral cancer survival rates [5]. In our series, we also found a better 10-year survival rate associated with treatment by high-volume physicians.

The quality of the risk-adjustment technique in analyzing administrative information is an important issue. In the first part of this study, a Cox proportional hazard model was used to compare the effects of high volume versus low/medium volume on survival rate. We found treatment by high-volume physicians was significantly associated with lower adjusted hazard ratio for death. Patients treated by high-volume physicians were found to have a 40% lower risk of death after adjusting for comorbidities and other confounding factors. However, there was some difference in age and clinical condition between caseload groups. In the second part of our series, propensity score was used to stratify patients into five strata with similar propensity score in order to reduce the effect of selection bias on caseload groups [19-21]. Patients treated by high-volume physicians were found to have a 14% relative improvement in adjusted 10-year survival rate (*p *< 0.001).

Although NPC patients may be followed up in a team consisting of otolaryngologist, radiation oncologists, hematology oncologists, and radiologists, the cornerstone of treatment of NPC relied on the successful eradication of disease by radiotherapy. In order to explore the caseload effect of radiotherapy on NPC survival, we calculated the caseload volume of radiation oncologists. In agreement with previous volume-outcome studies, our results indicated that increased caseload of radiation oncologists is associated with improved outcomes after other factors.

Several hypotheses relating to the volume-outcome relationship have been proposed. The "practice makes perfect" concept suggests that increased caseload may help physicians or hospital staff improve the execution of treatment procedures, such as planning the radiation field and manipulation of the radioactive source of teletherapy units. The role of surgery in the treatment of NPC is limited, and carefully defining the planning target volume with the aid of CT or MRI images is important for radiotherapy or concurrent chemoradiotherapy in NPC. A high-volume team may be more adept at administering a radiation dose, with or without a booster dose, that balances the benefit of successful loco-regional control against the risk of radiation toxicity.

Previous study reported that high-volume physicians use effective treatment and strategies more often than do low-volume physicians [22]. In breast cancer series, high-volume surgeons adopted a multi-disciplinary approach whereas low-volume surgeons were less likely to interact with oncologists or attend multi-disciplinary meetings [23]. Use of multidisciplinary approaches may account for the better outcomes achieved by high-volume physicians. Possibly, low-volume physicians do not always follow the international guidelines for NPC treatment.

The "selective referral hypothesis" postulates that healthier patients or patients with early-stage disease tend to be referred to high-volume physicians. The referral system in Taiwan is weakly enforced, and people are free to choose any physician. Because official performance information to help consumers select healthcare providers is not available, patients choose physicians with better reputations or more successful physicians after consulting with their relatives and friends [4]. Selective referral bias may also result from the referral of more curable patients to high-volume physicians. Patients not seeking curative treatment or for whom curative treatment is not possible may continue to receive their care from low-volume physicians.

Our study revealed some issues that may be useful for policy makers. Research is needed to identify the differences in care and treatment strategy between low-, medium-, and high-volume physicians. In our study, nearly 33% of patients were treated by 7 high-volume radiation oncologists. The viewpoints of high-volume physicians may influence the development of effective protocols and practice guidelines for the majority of clinical situations. The treatment strategies of high-volume physicians should be analyzed and adopted throughout the country to improve survival rates.

Our study has several limitations. First, we could not assess the relationship of caseload to NPC stage because this information was not available from the database. However, Begg et al., using a SEER-Medicare linked database, reported that cancer stage and patient age were independent of caseload volume [24]. Instead of cancer-specific survival rates, overall survival rate was used, because it was not possible to determine cause-specific mortality based on the registry data. Previous study by Roohan et al. showed no significant difference between survival models for all-cause mortality and breast cancer mortality [25]. Given the robustness of the evidence and statistical analysis in this study, these limitations are unlikely to compromise our results.

In summary, our findings support the conclusion that provider volume affects survival outcome in NPC. Analysis using a Cox proportional hazard model and propensity score found an association between high-volume physicians and improved 10-year survival rate in patients with NPC. Analysis of the treatment strategies adopted by high-volume physicians may improve overall survival rate.

## Conflict of interest

The authors declare that they have no competing interests.

## Authors' contributions

LCC, CSH and HSK developed the ideas for these studies, performed much of the work, and drafted the manuscript. CSH, CP, LCC, HTT and HSK revised the manuscript. LMS, SYC, CP, CWY and LHY designed the study, managed and interpreted the data. LCC performed the statistical analysis. All authors read and approved the final manuscript.
